# Validation of a Novel Cutaneous Neoplasm Diagnostic Self-Efficacy Instrument (CNDSEI) for Evaluating User-Perceived Confidence With Dermoscopy

**DOI:** 10.5826/dpc.1004a88

**Published:** 2020-10-26

**Authors:** Kelly C. Nelson, Ashley E. Brown, Amanda Herrmann, Chloe Dorsey, Julie M. Simon, Janice M. Wilson, Stephanie A. Savory, Lauren E. Haydu

**Affiliations:** 1 Department of Dermatology, The University of Texas MD Anderson Cancer Center, Houston, TX, USA; 2 McGovern Medical School, The University of Texas Health Science Center at Houston, Houston, TX, USA; 3 Department of Pathology, The University of Texas Health Science Center at Houston, Houston, TX, USA; 4 Department of Surgical Oncology, The University of Texas MD Anderson Cancer Center, Houston, TX, USA; 5 Department of Dermatology, The University of Texas Medical Branch, Galveston, TX, USA; 6 Department of Dermatology, The University of Texas Southwestern Medical Center, Dallas, TX, USA

**Keywords:** dermoscopy, construct validation, early detection, melanoma, dermatology education, self-efficacy, validation study, medical image interpretation

## Abstract

**Background:**

Accurate medical image interpretation is an essential proficiency for multiple medical specialties, including dermatologists and primary care providers. A dermatoscope, a ×10–×20 magnifying lens paired with a light source, enables enhanced visualization of skin cancer structures beyond standard visual inspection. Skilled interpretation of dermoscopic images improves diagnostic accuracy for skin cancer.

**Objective:**

Design and validation of Cutaneous Neoplasm Diagnostic Self-Efficacy Instrument (CNDSEI)—a new tool to assess dermatology residents’ confidence in dermoscopic diagnosis of skin tumors.

**Methods:**

In the 2018–2019 academic year, the authors administered the CNDSEI and the Long Dermoscopy Assessment (LDA), to measure dermoscopic image interpretation accuracy, to residents in 9 dermatology residency programs prior to dermoscopy educational intervention exposure. The authors conducted CNDSEI item analysis with inspection of response distribution histograms, assessed internal reliability using Cronbach’s coefficient alpha (α) and construct validity by comparing baseline CNDSEI and LDA results for corresponding lesions with one-way analysis of variance (ANOVA).

**Results:**

At baseline, residents respectively demonstrated significantly higher and lower CNDSEI scores for correctly and incorrectly diagnosed lesions on the LDA (P = 0.001). The internal consistency reliability of CNDSEI responses for the majority (13/15) of the lesion types was excellent (α ≥ 0.9) or good (0.8≥ α <0.9).

**Conclusions:**

The CNDSEI pilot established that the tool reliably measures user dermoscopic image interpretation confidence and that self-efficacy correlates with diagnostic accuracy. Precise alignment of medical image diagnostic performance and the self-efficacy instrument content offers opportunity for construct validation of novel medical image interpretation self-efficacy instruments.

## Introduction

Accurate medical image interpretation is a diagnostic proficiency in almost every area of medical education. The validation of metrics that quantify confidence and skill in image interpretation is necessary to measure the impact of educational efforts.

A dermatoscope is a medical device that pairs a 10× magnifier with polarized light, facilitating a more complete visualization of skin structures not readily visible to typical clinical examination. Skilled interpretation of dermoscopic images has been shown to reduce both false positives and false negatives during melanoma screening examinations when compared to clinical (naked-eye) examination alone [1,2]. Nevertheless, dermoscopy education in dermatology residency programs offers opportunity for improvement: 38% of US dermatology residents receive no dermoscopy training, and those residents who do receive training average only 2 hours of educational exposure [3,4]. Our group leveraged Project ECHO (Extension for Community Healthcare Outcomes), a telementoring framework, to deliver the DERMatology Early Melanoma Diagnosis (DERM:END) educational intervention [5].

To evaluate our educational intervention, we developed, tested, and validated 2 precisely aligned and complimentary instruments: the Long Dermoscopy Assessment (LDA) and the Cutaneous Neoplasm Diagnostic Self-Efficacy Instrument (CNDSEI). A panel of pigmented lesion and dermoscopy experts developed both instruments in accordance with the Association for Medical Education in Europe guidelines for a successful questionnaire [6]. Next, we utilized pilot versions of the LDA and CNDSEI in the first year of the telementoring education intervention. Based on participant feedback, we revised the instrument questions and responses for clarity and administered the revised instruments in year 2 of the program. To validate the CNDSEI, we demonstrate the feasibility and internal consistency reliability of the instrument as well as construct validity, or the degree to which the instrument measures what it intends to measure.

## Methods

As a quality improvement initiative, per policy, the curriculum and instrument development efforts did not require formal supervision by our institutional review board, but did receive Quality Improvement Advisory Board oversight. We delivered our educational intervention and instruments in the 2018–2019 academic year to 9 academic dermatology residency programs participating in the DERM:EMD program: University of Texas Health Science Center at Houston/MD Anderson Cancer Center Houston, Texas; Baylor College of Medicine Houston, Texas; Baylor Scott & White Health Dallas, Texas; University of North Texas Health Dallas, Texas; University of Texas Southwestern Medical Center Dallas, Texas; University of Texas at Austin Dell Medical School Austin, Texas; University of Texas Medical Branch Health Galveston, Texas; University of Missouri School of Medicine Columbia, Missouri; and Texas Tech University Health Sciences Center, Lubbock, Texas.

The CNDSEI, developed by our team (Figure S1), included 52 total questions divided into 4 distinct types: (1) How comfortable are you with diagnosing the following conditions with naked eye examination (n=15 different lesion types); (2) How comfortable are you with diagnosing the following conditions with dermoscopic examination (n=15); (3) How comfortable are you with distinguishing between the following (two) diagnoses with naked eye examination (n=11); and (4) How comfortable are you with distinguishing between the following (two) diagnoses with dermoscopic examination (n=11). Answer options included a Likert scale ranging from 1 to 10 with 1 labeled as “low comfort” and 10 labeled as “high comfort.”

The LDA included 30 dermoscopic images that residents were asked to classify in 3 ways: (1) whether the lesion is malignant or benign, (2) whether the lesion is melanocytic or non-melanocytic, and (3) the corresponding exact lesion type from options presented based on the logic of responses to #1 and #2 (Figure S2). Twenty-nine of the images corresponded exactly to lesion types that were interrogated on the CNDSEI. Both the CNDSEI and the LDA were administered prior to dermoscopy educational intervention exposure via the online survey tool, Qualtrics (Qualtrics International Inc., Provo, UT, USA).

We constructed pre-curriculum CNDSEI histograms for all responses, per question, and per lesion types (benign, malignant, melanocytic, and non-melanocytic), and visually inspected the response distributions. We assessed the internal consistency reliability of baseline CNDSEI to 15 items (question type 2 above), responses with Cronbach’s coefficient alpha, including assessment of inter-item mean correlations. The construct validity of the CNDSEI was assessed with the one-way analysis of variance (ANOVA) between CNDSEI responses (reflecting participant dermoscopic interpretation confidence) and LDA responses (reflecting participant dermoscopic interpretation performance) for each of the 29 specific lesion types queried on both assessments. We utilized IBM SPSS Statistics version 24 (SPSS; Chicago, IL) and SAS version 9.4 (SAS; Cary, NC) to conduct our analyses.

## Results

**Figure 1:**
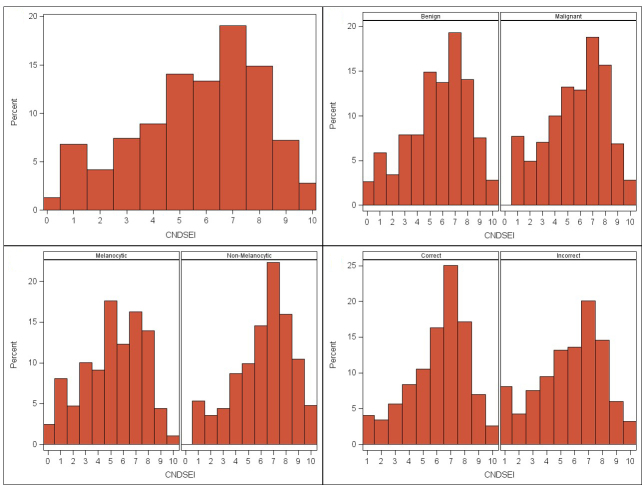
Distribution of baseline CNDSEI responses to 26 questions on the use of dermoscopy by 47 dermatology residents (n = 1,222 total responses): (A) overall, (B) by benign versus malignant lesion type, and (C) by melanocytic versus non-melanocytic lesion type. The Likert scale (1–10) was utilized with 1 defined as “low comfort,” 10 defined as “high comfort,” and 0 for a question not answered by a resident (no response).

**Figure 2:**
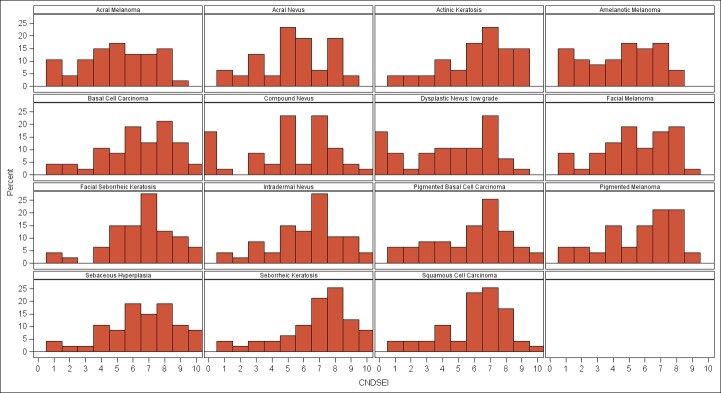
Distribution of baseline CNDSEI responses from 47 dermatology residents by question/lesion type. The Likert scale (1–10) was utilized with 1 defined as “low comfort,” 10 defined as “high comfort,” and 0 for a question not answered by a resident (no response).

Forty-seven dermatology residents completed the CNDSEI and LDA prior to receiving the 2018–2019 dermoscopy educational intervention. Generally, respondents accessed the survey without issues, understood what the questions asked, and finished the questionnaire in a timely manner, confirming instrument feasibility. Respondents utilized all response options on the 10-point Likert scale (1–10) (Figures 1 and 2). Eight of the 47 residents did not respond to the final 2 CNDSEI questions, and these are recorded as “0” on the histogram (n = 16).

The internal consistency reliability of 47 participant responses to 15 baseline CNDSEI items (diagnosing lesion types with dermoscopy) was excellent (α = 0.971), however, the mean inter-item correlation was high (0.712; range=0.335–0.961).

**Figure 3:**
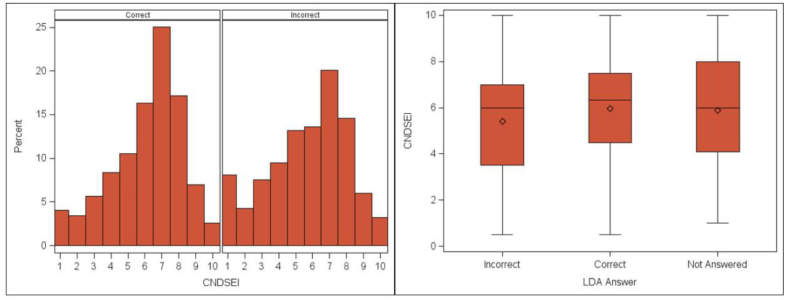
Distribution of CNDSEI responses to 15 “diagnosing with dermoscopy” questions according to the LDA result (correct or incorrect) for the corresponding lesion type (n = 1303). The Likert scale (1–10) was utilized with 1 defined as “low comfort,” 10 defined as “high comfort,” and 0 for a question not answered by a resident (no response).

To assess construct validity of the CNDSEI instrument, we examined the distribution of participant baseline CNDSEI responses (confidence) according to their LDA responses (accuracy) for corresponding lesion types (Figure 3). At baseline, residents demonstrated higher confidence (median 6.3, average 5.9 CNDSEI) for corresponding lesion types that they correctly identified on the LDA and lower confidence (median 6, average 5.4 CNDSEI) for corresponding lesions that were incorrectly identified on the LDA. There was a statistically significant difference in baseline CNDSEI scores for the lesion-types classified by participants on LDA correctly versus incorrectly (F = 6.91, P = 0.001).

## Discussion

The growing emphasis on competency-based medical education necessitates the validation of metrics that can quantify skill acquisition. Medical image interpretation skills are critical proficiencies in almost all fields of medical practice. In radiology there have been efforts to develop and validate simulation-based assessments that mirror real-life clinical decision-making [7] and to quantify radiographic image interpretation skills of non-radiologists [8]. Both tools relied on comparison of non-experts (medical students/interns) to more experienced users (senior residents) for construct validation of medical image interpretation accuracy [7,8].

While medical image interpretation accuracy is important, one’s perceived ability to achieve certain attainments [9], or self-efficacy, is an important determinant of practice change. Instruments addressing self-efficacy must be carefully validated to demonstrate the ability to appropriately capture user confidence in the construct it intends to measure [10]. We present a unique model for construct validation of medical image interpretation self-efficacy instruments by precisely aligning medical image interpretation accuracy and self-efficacy instrument content.

In validating an educational metric, it is important to meet 3 criteria: feasibility, reliability, and validity. Feasibility is the extent to which an instrument is simple, easy to understand, and brief. The best instruments are useless if they are incomprehensible, lengthy, and expensive. Pilot studies are used to test feasibility. Based on participant feedback from the initial CNDSEI pilot study, the authors reworded and reorganized questions for clarity. With the updated CNDSEI version, as validated herein, participants completed the instrument in a timely fashion, and demonstrated normal distribution of responses.

Reliability reflects the degree an instrument measures its endpoint accurately and is evaluated by examining the proportion of total variance that can be attributed to true differences between subjects. This is accomplished by the use of Cronbach’s coefficient alpha for intra-subject (one resident’s internal consistency between each question) and inter-subject (variability of residents’ responses per single question) reliability [10]. Our tool demonstrated excellent internal consistency reliability for intra-resident responses. Thus, we demonstrated that the overall confidence with dermoscopy was consistent for each resident. However, inter-item correlation was high, indicating that there is a limited aspect of the confidence construct being measured by each item. In addition, high levels of the Cronbach’s coefficient alpha can be a result of a large number of items, as required in our study to appropriately represent the range of cutaneous neoplasms encountered in dermatology practice.

There are many aspects of validity: face validity, content validity, and construct validity. The validation of an instrument should address all aspects of validity if possible. Face validity refers to the degree that an instrument appears to measure what it wants to measure, an important feature as subjects are less likely to answer questions that do not seem relevant. Content validity addresses whether the instrument covers most or all dimensions of the concept to be measured [10]. The analysis establishing content validity is theoretical and based on expert opinion, systematic review of the literature review, and factor analysis. Factor analysis is the grouping of questions into subtypes to meet each desired domain. In our tool, questions were grouped by lesion type and more broadly by benign and malignant types.

Finally, construct validity, classically inferred by the term validity, is the degree to which an instrument measures what it intends to measure. Construct validity is evaluated by exploring the relation of the instrument with the behavior it measures [10].

Our study offers proof of concept that close alignment of the medical image interpretation accuracy instrument and the self-efficacy instrument content facilitates construct validation of the self-efficacy instrument, a concept that is applicable to other educational settings, including pathology and radiology. In our tool, confidence in dermoscopy skill with specific lesion types was directly and significantly related to the ability to correctly diagnose those same lesion types on the LDA.

## Limitations

Eight of the 47 residents did not reach the final 2 questions; participant feedback on this limitation included both timing and patchy internet signaling at their testing sites. In future versions more time will be allotted for use if needed. Questionable inter-observer reliability for compound nevus can be attributed to the low number of questions.

Additionally, this instrument is limited by being validated against 1 specific dermoscopy assessment, the LDA. The LDA was designed with questions specific to the didactic content in the author’s (K.C.N.) dermoscopy curriculum, which emphasized distinguishing between specific pairings of lesions with similar clinical but distinct dermoscopic features, an educational approach that may not be shared by other curricula. This could alter the validity of the self-efficacy instrument when compared to another dermoscopy assessments, especially in regard to the “distinguishing between” questions.

Finally, inter-rater and test-retest reliability were not assessed. Test-retest reliability was not feasible to assess, as we only tested at baseline and after intervention with the intention of seeing a variance. Inter-rater reliability is not applicable to the construct in this context, as we are measuring self-perceived confidence that cannot be quantified by a separate rater.

## Conclusions

Skillful interpretation of dermoscopic images offers an opportunity to reduce false negatives and positives in the detection of melanoma. Validation of instruments to quantify dermoscopic skill and confidence is essential for the development and iterative improvement of sound dermoscopy educational programs.

The CNDSEI instrument can accurately measure confidence in dermoscopic skill and has predictive value for the ability to diagnose lesions correctly with dermoscopy. The validation of this tool will be useful in measuring change in dermoscopy education interventions for both dermatology and primary care residents. Supporting performance of full-skin examinations and skillful dermoscopic evaluation of lesions of concern by primary care providers in low dermatology access areas offers the opportunity to reduce diagnostic barriers in broader patient populations. Additionally, the concept of aligning medical image interpretation and self-efficacy instrument content can be broadly applied to medical image interpretation fields beyond dermatology, including radiology, pathology, gynecology, and others. The ability to accurately quantify medical image interpretation skill acquisition has practical application across many fields in patient care.

## Supplementary Information

Figure S1 The Cutaneous Neoplasm Dermoscopy Self-efficacy Instrument (CNDSEI).

Figure S2 The Long Dermoscopy Assessment (LDA) question format and branching logic
