# Supplementary figures and images for: Validation of a Novel Cutaneous Neoplasm Diagnostic Self-Efficacy Instrument (CNDSEI) for Evaluating User-Perceived Confidence With Dermoscopy
Source: Dermatol Pract Concept. 2020 Oct 26;10(4):e2020088. doi: 10.5826/dpc.1004a88 (PMC7588154; doi:10.5826/dpc.1004a88)

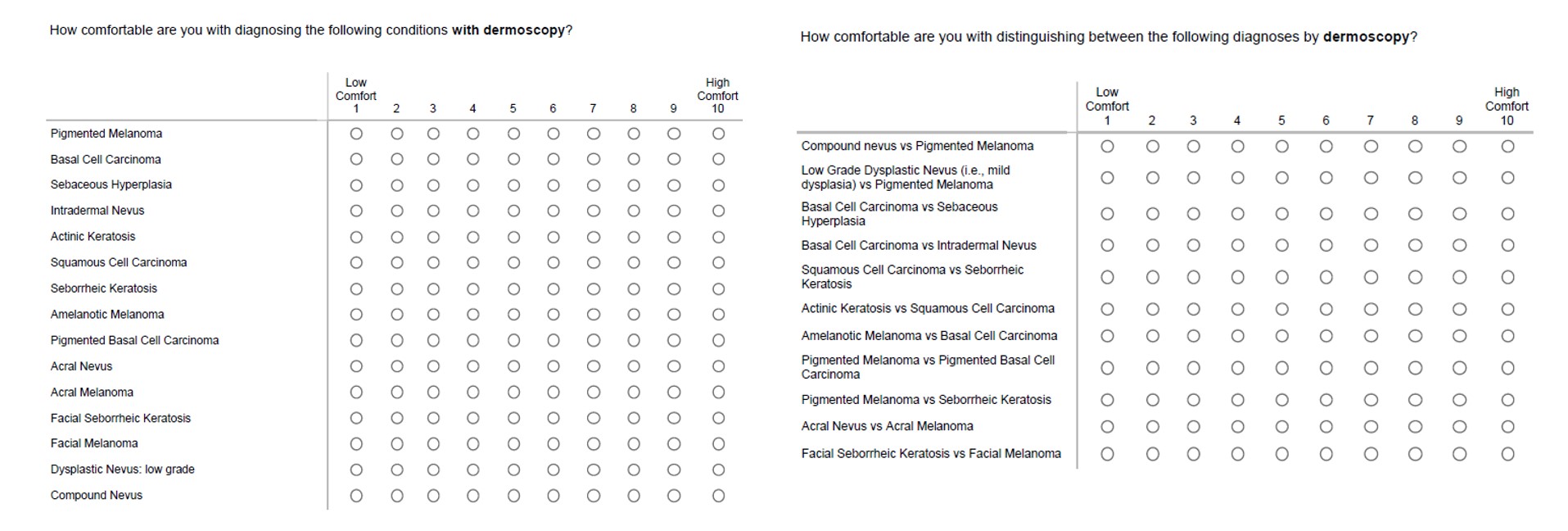

Supplement: Figure S1 — The Cutaneous Neoplasm Dermoscopy Self-efficacy Instrument (CNDSEI). [file dp1004a88g00s1.jpg]

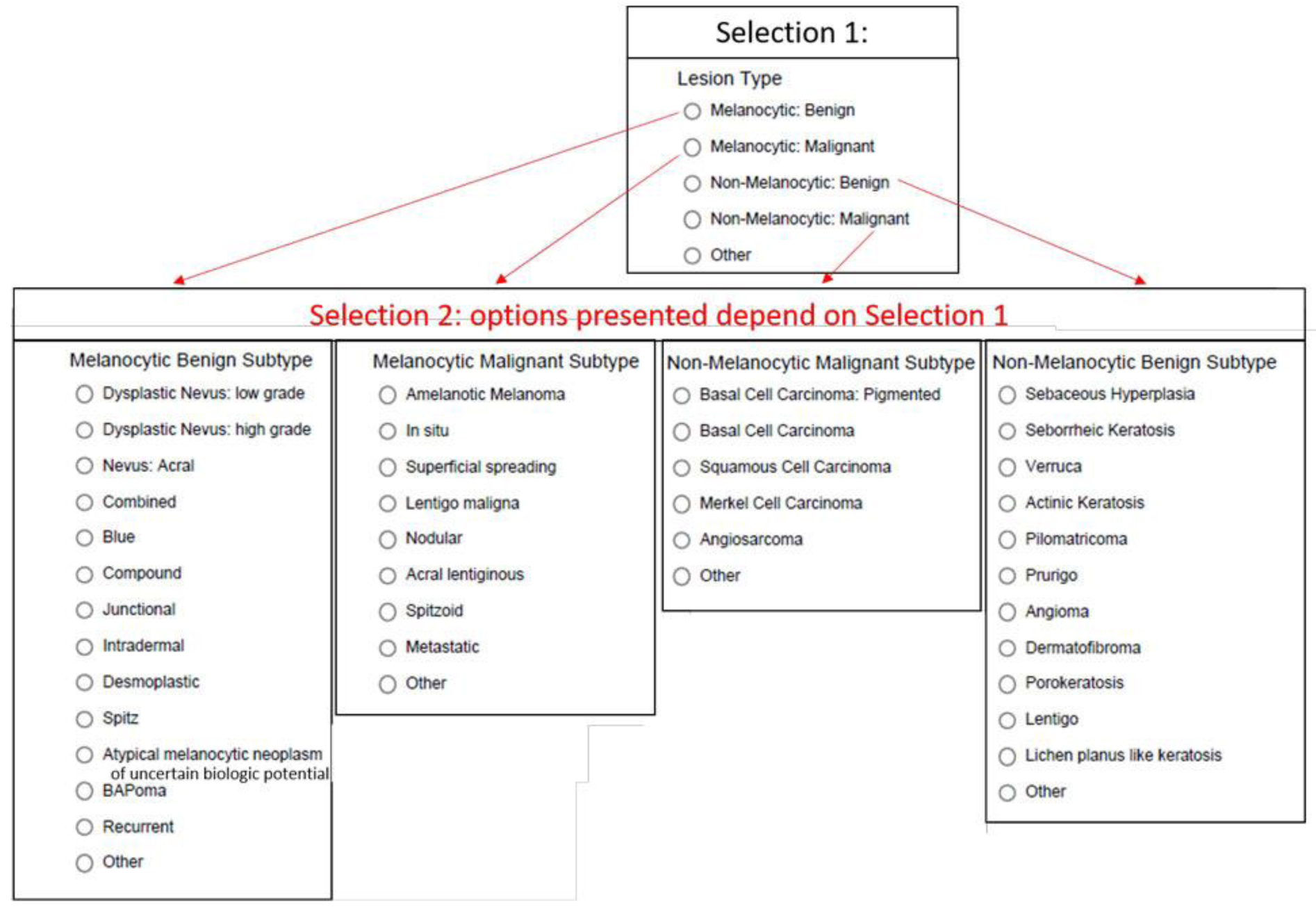

Supplement: Figure S2 — The Long Dermoscopy Assessment (LDA) question format and branching logic [file dp1004a88g00s2.jpg]
